# Randomized controlled trial to assess medication adherence and health-related quality of life through a collaborative pharmacist-psychiatrist approach to patient education in patients with depression in India

**DOI:** 10.3389/fpsyt.2025.1499893

**Published:** 2025-02-25

**Authors:** Ambed Mishra, Manohar Rao Kishor, Madhan Ramesh

**Affiliations:** ^1^ Department of Community Medicine, JSS Medical College and Hospital, JSS Academy of Higher Education & Research, Mysore, Karnataka, India; ^2^ Department of Psychiatry, JSS Medical College and Hospital, JSS Academy of Higher Education & Research, Mysore, Karnataka, India; ^3^ Department of Pharmacy Practice, JSS College of Pharmacy, JSS Academy of Higher Education & Research, Mysore, Karnataka, India

**Keywords:** depression, medication adherence, quality of life, pharmacy, psychiatry, mental health

## Abstract

**Background:**

Depression is a common but severe mental health disorder affecting individuals globally. Medication non-adherence and low health-related quality of life (HRQoL) are the major challenges associated with the treatment of patients with depression.

**Materials and methods:**

A prospective Randomized Controlled Trial (RCT) was conducted in the psychiatry outpatient department of a tertiary care hospital for six months. Patients were assigned to either a control group receiving usual care or a test group receiving collaborative care using simple randomization technique. The eligible patients diagnosed with depression were enrolled and the data were collected from patient’s care records, prescriptions, patient interviews, patient representatives and healthcare professionals. The intervention in the test group consisted of comprehensive patient education, including a thorough counseling session with a research clinical pharmacist. Counseling sessions included information on the disease, medications prescribed, possible side effects, compliance with medications and overall treatment. The data collected from both patient groups was analyzed for medication adherence using the Medication Adherence Rating Scale (MARS) and health-related quality of life (HRQoL) using the WHOQOL-BREF questionnaire. Statistical analyses were performed using a student t-test with a significance level of P value < 0.05.

**Results:**

The collaborative care group showed a statistically significant improvement in medication adherence, with a mean increase of 1.67 ± 0.25 (P < 0.001), compared with a mean increase of 0.69 ± 0.05 (P < 0.05) for the usual care group. Similarly, HRQoL scores also improved significantly more in the collaborative care group, with a mean increase of 28.01 ± 2.05 (P < 0.001), compared with a mean increase of 12.46 ± 0.26 (P < 0.05) for the usual care group.

**Conclusion:**

This study concluded that pharmacist–psychiatrist collaborative patient education can significantly improve the medication adherence and HRQoL of the patients with depression. Statistically significant increases in medication adherence and HRQoL were observed in the collaborative group.

## 1 Introduction

Depression is a prevalent mental disorder characterized by loss of interest or pleasure in daily activities, feelings of low self-esteem, disturbed sleep or appetite, unusual tiredness and poor concentration. It can be prolonged or recurrent, significantly diminishing an individual’s ability to function and cope with daily life (1). In its acute stage, depression can sometimes be managed without medications through various coping methods, however, when depression becomes severe and poses risks to life, patients may require pharmacological interventions. Depression can be diagnosed with and treated by non-specialists within the primary healthcare team. Specific care is only necessary for individuals with complicated depression or those who do not respond to first-line treatments (2).

People taking psychotropic medications for mental health disorders including depression are at high risk of experiencing medication-related problems. Medication non-adherence and treatment discontinuation are the major challenges faced by mental health professionals, however, educating the patients helps mitigate such issues. Healthcare providers, including physicians, pharmacists and nurse practitioners, can enhance the effectiveness and efficiency of care by providing specific education on diseases and medications. Clinical Pharmacists, as medical experts, play an important role in disease and medication counseling, which is a valuable source of information for patients and their caregivers (3, 4).

Various studies where the clinical pharmacist was a collaborative care team member for patient care in either hospital or community set-up, have shown that patient education provided by pharmacists which includes information regarding the disease and medications, results in improved health outcomes for medication adherence with quality of life, when compared to the traditional care approach in India with the physician as the sole member (3–7).

Medication adherence plays a crucial role in the effective treatment of all diseases, including depression. Adhering to the prescribed medication regimen is essential for patients to improve their health-related quality of life (HRQoL). HRQoL is an overall indicator of how diseases and their treatment affect a person’s overall functioning and well-being. It encompasses physical, psychological and social aspects of health, reflecting both the positive and negative effects on a person’s ability to lead a fulfilling life (6, 7).

Hence, this study aimed to initiate and assess the impact of the pharmacist-psychiatrist collaborative patient education model on medication adherence and the health-related quality of life in patients with depression.

## 2 Materials and methods

### 2.1 Study design

A prospective Randomized Controlled Trial (RCT) was conducted in the outpatient department of Psychiatry at a tertiary care teaching hospital in South India for 6 months. The study received ethical approval from the Institutional Human Ethics Committee of JSS Academy of Higher Education & Research (formerly known as JSS University), Mysore, Karnataka, India. This study is a continuation of ongoing research focused on understanding psychiatric issues and exploring multi-department collaborative care interventions that can improve medication adherence and the overall health-related quality of life for patients in India (5, 6).

The study included patients who visited the psychiatry outpatient department and were of either gender, aged ≥18 years, treated for depression and were literate. Literacy was assessed by interviewing patients regarding their education which included basic reading and writing. The illiterate patients were excluded from our study. Literacy was an inclusion criterion because the patients needed to answer the questionnaires for each follow-up.

Patients with pre-existing comorbidities, including other psychiatric conditions such as cognitive dysfunction or significant illnesses, were excluded from the study. All patients underwent detailed screening for any known history of diseases, with an additional focus on individuals aged 60 or older due to the higher prevalence of cognitive dysfunction in this age group. This comprehensive screening process ensured that only those with newly diagnosed depression were included in the study, excluding anyone with a history of other conditions. Similarly, patients receiving treatment in ambulatory care settings in other departments were not included. Relevant and necessary data for evaluation were collected from outpatient case records, patient prescriptions, communication with healthcare professionals, patient and caretaker interviews.

### 2.2 Study procedure

Patients with depression who met the study criteria were included in the study in consultation with a psychiatrist after obtaining informed consent from the patients. The enrolled subjects were grouped into test (collaborative care) and control (regular or usual care) groups using a simple randomization technique where the psychiatrists who provided usual care to the patients in the hospital were blinded i.e., were unaware of the patients’ groups to ensure the same treatment is provided to each patient by the psychiatrist irrespective of the test or control groups where patients were randomized.

Patient education was provided to the test group by the clinical pharmacist and included verbal counseling along with a specially designed Patient Information Leaflet (PIL) for depression in English and translated to the local language Kannada by the local government-approved linguistic expert, to aid the patient’s understanding of their disease condition & prescription. The patient education was customized for each case based on the severity of the disease and involved answering questions from patients or their careers. Patient education sessions by the research clinical pharmacist covered information about the disease, prescribed medications, potential side effects, the importance of medication adherence and the overall treatment regimen to help the patient understand their condition better.

Usual care by psychiatrists involved an examination of the patient’s disease and prescribing medications during the patient consultation session. The patients of both test and control groups were followed-up for a total of three sessions including the initial enrollment at the time of diagnosis and then during each follow-up which was as per the patient’s regular date of next visit as per the psychiatrist. The research pharmacist called the patients at least one week and then a day before their next scheduled hospital visit date to ensure that the patients did not miss their next follow-up.

The patient education session addressing the concerns of the patients and providing counseling to the carers including the family members of the patients was done during follow-ups, to ensure better medication adherence and health-related quality of life with improved overall treatment results were achieved for the patients in the test group.

The control group patients’ medication adherence & health-related quality of life were also monitored during follow-ups to compare the results with traditional approach to treatment by psychiatrist.

Medication adherence and health-related quality of life were assessed for both the test and control group patients during each visit to the psychiatrist followed by a session with the research clinical pharmacist for all patients. Scales for assessing the medication adherence & health-related quality of life were administered to all the patients with specific patient education and counseling provided only to the test group patients by the research clinical pharmacist.

#### 2.2.1 Scales and psychometric properties

##### 2.2.1.1 Medication adherence rating scale

Medication adherence was evaluated using the Medication Adherence Rating Scale (MARS) questionnaire in both English and the local language Kannada translated by a government-approved local language Kannada-linguistic expert and validated by a team of pharmacists and psychiatrists. MARS is a validated tool, highly reliable in the assessment of adherence; it has higher scores indicating better medication compliance. The MARS is a ten-item scale rated on a binary scale reflecting the patient’s attitude and behavior toward his or her prescribed medication regimen. The MARS scale is well-documented for its reliability and validity, with higher scores indicating better adherence. The MARS questionnaire helps healthcare providers understand patients’ attitude about medication so they can offer better support (8).

##### 2.2.1.2 The World Health Organization quality of life-BREF scale

The WHOQOL-BREF assesses the quality of life across four domains: Physical Health, Psychological Health, Social Relationships and Environmental Quality. Scores for each domain are normalized to a scale from 0 to 100, with higher scores reflecting better outcomes. Its psychometric properties, including internal consistency and validity, have been widely tested in various populations. The WHOQOL-BREF questionnaire includes 26 questions and looks at quality of life in four main areas in four sections, also known as domains. The Physical Health domain assesses things like energy, mobility, pain, sleep and how well someone can handle daily tasks. The Psychological Health domain focuses on emotions, including enjoyment, satisfaction with life, concentration, self-esteem, body image and feelings like anxiety or depression. The Social Relationships domain looks at personal relationships, support from others and satisfaction with sexual life. The environmental domain includes questions about safety, access to healthcare, finances, living conditions, leisure activities and the quality of things like transportation and pollution. These areas together with 26 questions help to understand a person’s overall health-related quality of life compared to various sections or domains of life (9). Quality of life was assessed using the World Health Organization Quality of life-BREF (WHOQOL-BREF) questionnaire which was used in English with its officially translated version available from the Information, Evidence and Research (IER) Department of The World Health Organization (WHO), Geneva, Switzerland provided to us for our research as per our request.

Written permissions to use the MARS and WHOQoL questionnaires were obtained by researcher & author Dr. Ambed Mishra from Dr. Katherine Thompson, Mitcham, Victoria, Australia, the original author and scientist of the Medication Adherence Rating Scale (MARS) and the Information, Evidence and Research (IER) Department of The World Health Organization (WHO), Geneva, Switzerland, respectively.

### 2.3 Statistical analysis

The collected data were analyzed using SPSS version 20. Descriptive statistics on sample characteristics were computed, including means, standard deviation and frequency distributions. Differences between means were calculated using a t-test and P-values < 0.05 were considered statistically significant. Means are expressed as Mean ± Standard Deviation.

## 3 Results

### 3.1 Patient demographics

A total of 75 patients diagnosed with depression who met the study criteria were enrolled and all the patients completed the study. Thirty-six patients were in the test group and thirty-nine patients were assigned to the control group. The majority of study participants were aged 18–39 years (58.67%), while only 2.67% were aged 60 years or older, with a mean age of 38.57 ± 12.37 years. Most of the patients who completed the study were female (65.34%). Demographic details of the study patients are presented in **Table 1**.

**Table 1:** Demographic details of the study population.

Demographics	Category	No. of Patients (n = 75)	Percentage (%)	Usual care (n=39)	Collaborative care (n=36)
**AGE**	18-39	44	58.67%	25	19
	40-59	25	33.34%	12	13
	≥ 60	6	2.67%	2	4
	Mean ***±*** SD	38.57 ***±*** 12.37	100%	36.11 ***±*** 11.75	41.25 ***±*** 12.64
**GENDER**	Male	26	34.67%	11	15
	Female	49	65.34%	28	21
**MARITAL STATUS**	Single	18	24%	10	8
	Married	57	76%	29	28

### 3.2 Assessment of medication adherence

Medication adherence was assessed using the Medication Adherence Rating Scale (MARS), a reliable and validated standard tool with a Cronbach’s alpha of 0.75–0.85. Higher MARS scores indicate better adherence.

#### 3.2.1 Usual care group follow-ups

Assessment of the patient’s medication adherence to the usual care from 1^st^ follow-up to 2^nd^ follow-up and from 2^nd^ follow-up to 3^rd^ follow-up showed a mean increase in medication adherence levels of 0.38 ± 0.01 and 0.31 ± 0.04, respectively. The overall mean increase in medication adherence (from the 1^st^ follow-up to the 3^rd^ follow-up) in the usual care group was found to be 0.69 ± 0.05. Most of the values were statistically significant (P-value < 0.05) as shown in **Table 2**.

**Table 2:** Comparison of medication adherence scores among usual care group follow-ups.

FOLLOW-UPs	(Mean ± S.D)	(Mean ± S.D)	P-VALUE*
1^st^ Vs 2^nd^ Follow-Up	5.38 ± 0.78 (1^st^)	5.76 ± 0.77 (2^nd^)	0.032**
2^nd^ Vs 3^rd^ Follow-Up	5.76 ± 0.77 (2^nd^)	6.07 ± 0.73 (3^rd^)	0.77
1^st^ Vs 3^rd^ Follow-Up	5.38 ± 0.78 (1^st^)	6.07 ± 0.73 (3^rd^)	<0.001**

*T-test; **statistically significant.

#### 3.2.2 Collaborative care group follow-ups

Similarly, the assessment of patient’s medication adherence to collaborative care from 1^st^ follow-up to 2^nd^ follow-up and from 2^nd^ follow-up to 3^rd^ follow-up showed a mean increase in medication adherence levels of 0.92 ± 0.6 and 0.75 ± 0.19, respectively. The overall mean increase in medication adherence (from 1^st^ follow-up to 3^rd^ follow-up) in the usual group was found to be 1.67 ± 0.25. All the values were statistically significant (P-value < 0.001), as shown in **Table 3**.

**Table 3:** Comparison of medication adherence scores among Collaborative care group follow-ups.

FOLLOW-UPs	(Mean ± S.D)	(Mean ± S.D)	P-VALUE*
1^st^ Vs 2^nd^ Follow-Up	5.30 ± 0.62 (1^st^)	6.22 ± 0.68 (2^nd^)	<0.001**
2^nd^ Vs 3^rd^ Follow-Up	6.22 ± 0.68 (2^nd^)	6.97 ± 0.87 (3^rd^)	<0.001**
1^st^ Vs 3^rd^ Follow-Up	5.30 ± 0.62 (1^st^)	6.97 ± 0.87 (3^rd^)	<0.001**

*T-test; **statistically significant.

#### 3.2.3 Comparison between usual care and collaborative care groups

The usual care group showed significant improvement between the 1st and 2nd follow-up (P = 0.032**) and the 1st and 3rd follow-up (P < 0.001**), while no significant change occurred between the 2nd and 3rd follow-up (P = 0.77).

**Figure 1:**
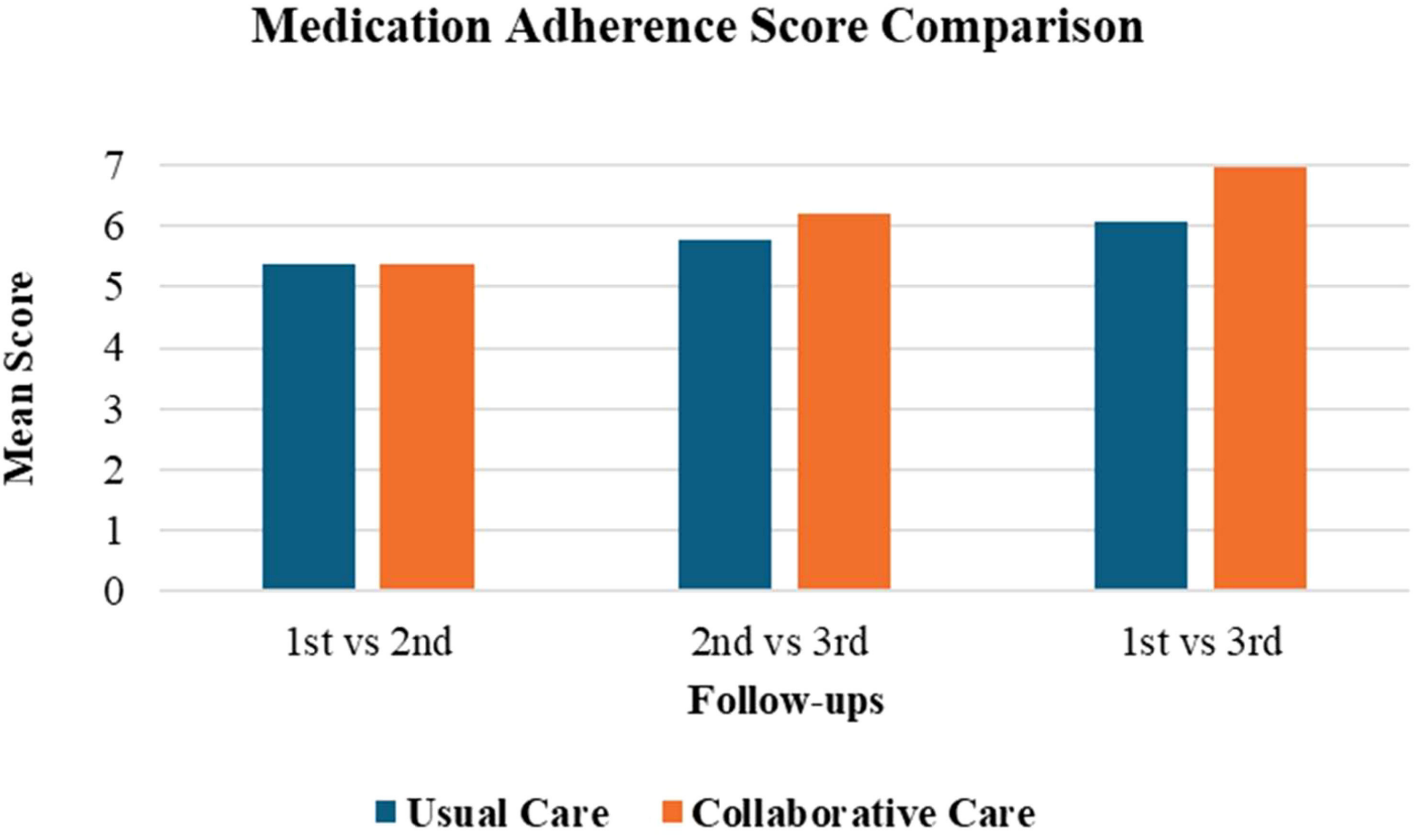
Medication adherence score comparison between the two groups.

The mean increase in medication adherence from baseline to the final follow-up in the collaborative care group was 1.67 ± 0.25, which was statistically significant (P < 0.001) as illustrated in **Figure 1**. The usual care group presented a mean increased value of 0.69 ± 0.05, with P < 0.05. Comparing both groups directly, it was determined that the collaborative care group provided an additional improvement over usual care of 0.98 ± 0.20 and was more effective in improving the medication adherence outcomes of the patient. Adding a psychiatry-trained clinical pharmacist to provide patient education and resolve any doubts from patients or carers improved medication adherence compared to care provided solely by a psychiatrist. This also allowed psychiatrists to manage more patients, with clinical pharmacists playing a key role in improving adherence.

#### 3.2.4 Significance of collaborative care in improving medication adherence

The collaborative care approach, which included a psychiatry-trained clinical pharmacist alongside the psychiatrist, showed overall better results and improvements than the usual care in increasing medication adherence. From the baseline to the final follow-up, the collaborative care group showed a mean improvement of 1.67 ± 0.25 (P < 0.001), which was significantly higher than the 0.69 ± 0.05 (P < 0.05) improvement in the usual care group. This difference of 0.98 ± 0.20 highlights how much of an impact the clinical pharmacist had in answering patients’ and caregivers’ questions and providing personalized patient education with the counseling sessions. It also allowed psychiatrists to treat more patients, making collaborative care not only more effective but also a more efficient way to improve patient outcomes.

### 3.3 Assessment of quality of life

HRQoL was assessed using the WHOQOL-BREF, with higher scores indicating better health-related quality of life across four domains: Physical, Psychological, Social and Environmental.

#### 3.3.1 HRQoL in usual care group follow-ups

The assessment of the patient’s health-related quality of life of the usual care group from 1^st^ follow-up to the 2^nd^ follow-up, from 2^nd^ follow-up to the 3^rd^ follow-up and from 1^st^ follow-up to 3^rd^ follow-up showed mean increases of 11.41 ± 0.49 and 1.05 ± 0.23 and 12.46 ± 0.26 respectively. The comparison of QOL scores between usual care and collaborative care is shown in **Table 4**.

**Table 4:** Comparison of WHOQOL scores among usual care group follow-ups.

Follow-up	Domains	(Mean ± S. D.)	(Mean ± S. D.)	P value* (<0.05)
1^st^ Vs 2^nd^	Domain 1	28.87 ± 10.46	41.94 ± 8.36	<0.001**
	Domain 2	25.92 ± 14.53	42.10 ± 9.5	<0.001**
	Domain 3	36.33 ± 5.76	42.02 ± 11.34	0.007
	Domain 4	34.48 ± 7.84	45.2 ± 11.35	<0.001**
	**Overall score**	**31.4**± **9.64**	**42.81 ± 10.13**	–
2^nd^ Vs 3^rd^	Domain 1	41.94 ± 8.36	42.28 ± 8.15	0.859
	Domain 2	42.10 ± 9.5	42.66 ± 10.64	0.806
	Domain 3	42.02 ± 11.34	43.92 ± 13.33	0.501
	Domain 4	45.2 ± 11.35	46.61 ± 7.49	0.52
	**Overall score**	**42.81 ± 10.13**	**43.86 ± 9.9**	–
1^st^ Vs 3^rd^	Domain 1	28.87 ± 10.46	42.28 ± 8.15	<0.001**
	Domain 2	25.92 ± 14.53	42.66 ± 10.64	<0.001**
	Domain 3	36.33 ± 5.76	43.92 ± 13.33	0.002
	Domain 4	34.48 ± 7.84	46.61 ± 7.49	<0.001**
	**Overall score**	**31.4**± **9.64**	**43.86 ± 9.9**	**–**

*T-test; **statistically significant.

Domain 1- Physical Health Domain 2- Psychological Health.

Domain 3- Social Relationships Domain 4- Environmental Quality of Life.

Bold values: Overall scores for each category.

#### 3.3.2 HRQoL in collaborative care group follow-ups

The assessment of the patient’s health-related quality of life of the collaborative care from 1^st^ follow-up to 2^nd^ follow-up, from 2^nd^ follow-up to 3^rd^ follow-up and from 1^st^ follow-up to 3^rd^ follow-up showed mean increases of 11.84 ± 0.46 and 16.17 ± 1.59 and 28.01 ± 2.05 respectively. The comparison of HRQoL scores between usual care and collaborative care is shown in **Table 5**.

**Table 5:** Comparison of WHOQOL scores among Collaborative care group follow-ups.

Follow-up	Domains	(Mean ± S. D.)	(Mean ± S. D.)	P value* (<0.05)
1^st^ Vs 2^nd^	Domain 1	35.36 ± 13.02	51.25 ± 9.67	<0.001**
	Domain 2	31.97 ± 12.82	52.33±8.11	<0.001**
	Domain 3	38.61 ± 10.54	52.97 ± 10.81	<0.001**
	Domain 4	40.72 ± 8.16	54.75 ± 9.59	<0.001**
	**Overall score**	**36.65** ± **11.13**	**52.82 ± 9.54**	<0.001**
2^nd^ Vs 3^rd^	Domain 1	51.25 ± 9.67	60.58 ± 9.91	<0.001**
	Domain 2	52.33±8.11	67.08 ± 8.88	<0.001**
	Domain 3	52.97 ± 10.81	65.86 ± 9.2	<0.001**
	Domain 4	54.75 ± 9.59	65.13 ± 8.33	<0.001**
	**Overall score**	**52.82 ± 9.54**	**64.66 ± 9.08**	<0.001**
1^st^ Vs ^3rd^	Domain 1	35.36 ± 13.02	60.58 ± 9.91	<0.001**
	Domain 2	52.33±8.11	67.08 ± 8.88	<0.001**
	Domain 3	38.61 ± 10.54	65.86 ± 9.2	<0.001**
	Domain 4	40.72 ± 8.16	65.13 ± 8.33	<0.001**
	**Overall score**	**36.65±11.13**	**64.66 ± 9.08**	<0.001**

*, T-test; **, statistically significant.

Domain 1, Physical Health; Domain 2, Psychological Health; Domain 3, Social Relationships; Domain 4, Environmental Quality of Life.

Bold values indicate the overall score of each sub-section.

#### 3.3.3 Comparison of HRQoL between usual care and collaborative care groups

Patients in the collaborative care group experienced a mean increase of 28.01 ± 2.05 in HRQoL scores across the four domains (Physical, Psychological, Social and Environmental) by the final follow-up, which was highly significant (P < 0.001). The usual care group, however, showed a more modest improvement of 12.46 ± 0.26 (P < 0.05). The collaborative care intervention contributed an additional improvement of 15.55 ± 1.79 in HRQoL scores when compared to the usual care group. In the collaborative care group, all domains of HRQoL (Physical, Psychological, Social and Environmental) showed consistent and statistically significant improvements compared to the usual care group.

The overall health-related quality of life improved in the test group receiving collaborative care from both a psychiatrist and a research clinical pharmacist, compared to the control group receiving usual care solely from a psychiatrist.

#### 3.3.4 Significance of collaborative care in improving HRQoL

Including a psychiatry-trained clinical pharmacist in the collaborative care model resulted in a much larger improvement in patients’ quality of life compared to the usual care group. The collaborative care group showed a mean increase of 28.01 ± 2.05 (P < 0.001), with significant improvements in all four HRQoL domains (Physical, Psychological, Social and Environmental). In comparison, the usual care group had a more modest improvement of 12.46 ± 0.26 (P < 0.05), highlighting the added benefit of the collaborative care approach. The difference of 15.55 ± 1.79 in HRQoL scores highlights the value of combining the expertise of both a psychiatrist and a clinical pharmacist, ultimately providing better overall care for patients. These results show how collaborative care can make a real difference in improving patients’ health-related quality of life.

## 4 Discussion

The average increase in medication adherence was 0.98 ± 0.2 when comparing the collaborative care group to the usual care group, indicating a better medication adherence outcome in the collaborative care (test) group. Similarly, the assessment of patients’ HRQoL between both groups showed a mean increase of 15.55 ± 1.79.

This study seems to be the first of its kind in India to use a pharmacist-psychiatrist collaborative patient education model to assess the impact of medication adherence and HRQoL for patients diagnosed with depression in a tertiary care setting. The outcomes of this study are similar to those in the study by Capoccia et al. (9) for pharmacist interventions to improve depression care and outcomes in primary care, where the mean score improved in follow-ups. However, this study found statistically significant differences between the treatment and usual care groups. This may be due to the specially designed patient education leaflets and counseling sessions.

A similar study conducted in Iran demonstrated significant positive correlations between medication adherence and various domains of quality of life, such as emotional well-being, social functioning and general health, among patients with type 2 diabetes. This aligns with the findings of the current study, which also observed improved medication adherence leading to enhanced quality of life metrics. Additionally, the Iranian study highlighted the need for self-care education programs to address poor adherence, a recommendation that reinforces the potential benefits of collaborative care models like the one evaluated in this study (10).

The strengths of this study include its focus on key factors like medication non-adherence and decreased HRQoL in patients with depression. These factors can be improved and can have a positive impact on the overall health outcomes of patients. Most clinical pharmacy services were focused on inpatient hospital settings. Patients visiting outpatient departments of hospitals in India often do not receive adequate clinical pharmacist care due to the unavailability of competent pharmacists at all the hospitals in India (6, 11).

In India, clinical pharmacist-physician collaborative care is a relatively new concept. Though clinical pharmacist services were initiated nearly two decades ago in India, the involvement of clinical pharmacists in mental healthcare has been minimal (11). Clinical pharmacists with a Doctor of Pharmacy (PharmD, integrated doctoral degree), is a relatively new concept in India, modeled after the PharmD education system in the United States. In India, the PharmD program is a six-year integrated degree, while a three-year program, known as PharmD Post Baccalaureate (PharmD PB), is available for pharmacists who have completed a traditional four-year Bachelor of Pharmacy (BPharm) degree. The Pharmacy Council of India (PCI), based in New Delhi, regulates both the PharmD and PharmD PB programs and has established guidelines for their implementation (12).

Additionally, a selected few institutions in India like the JSS Academy of Higher Education & Research (JSS AHER) are accredited by the Accreditation Council for Pharmacy Education (ACPE), Chicago, Illinois in the United States which further affirms their adherence to international standards of pharmacy education (12). Both the six-year PharmD and the three-year PharmD PB programs provide extensive training with internships in community-based and hospital patient care, under the supervision of a team of senior Doctor of Medicine (MD) physicians. The introduction of the Doctor of Pharmacy (PharmD) program in India in 2008 has significantly expanded the role of clinical pharmacists as a collaborative care team member in Indian hospitals (12, 13).

Medications are prescribed to treat different ailments. Still, information given to patients on the proper use of these medications is minimal due to the heavy workload of healthcare professionals in outpatient departments. The provision of medication usage information by dispensing pharmacists to individuals with mental health issues is also minimal, leading to high rates of medical non-adherence, treatment discontinuation and related complications among Indian psychiatric patients (12–14). Lack of knowledge about psychotropic medications among dispensing pharmacists, unavailability of trained clinical, hospital and community pharmacists, busy work schedules and lack of inter-professional collaboration between pharmacists and psychiatrists may contribute to these limitations (4, 11).

This study did not include all the outpatients being treated for other co-morbidities and in other hospital departments. This might have resulted in a smaller sample size and an underestimation of the parameters like improvement in HRQoL and medication adherence due to the collaborative care approach. The study was limited to outpatients and did not include inpatients at the time of discharge, which limited the assessment of the pharmacist’s collaborative role in patients’ medication adherence and health-related quality of life in the inpatient setup.

Medication non-adherence and decreased HRQoL are major issues, especially in patients with mental health disorders like depression (6, 7). Hence, the initiation of a pharmacist-psychiatrist collaborative patient education model for patients with depression and its implementation for other diseases may prove beneficial in minimizing the issues related to medication non-adherence to a greater extent (6, 7, 15–17).

## 5 Limitations

- The study was conducted in a single hospital, limiting geographic and institutional diversity, which may affect the generalizability of the findings.
- The questionnaire-based assessments included only literate patients, excluding non-literate individuals and potentially biasing the sample against a significant portion of the target population.
- Medication adherence and health-related quality of life (HRQoL) were assessed in patient batches over a six-month period, restricting the scope of long-term or continuous observation.

## 6 Future suggestions

- Expanding this model into a multi-center study across multiple hospitals, regions and countries could produce more generalized and globally applicable results.
- Developing modified assessment tools, such as oral interviews or visual scales, could make future studies inclusive of non-literate populations, addressing the limitation of questionnaire-based assessments.
- Conducting long-term studies with follow-up periods extending over multiple years could provide more insights into medication adherence and the sustained effects of the intervention on HRQoL.

## 7 Conclusion

The study concludes that pharmacist-psychiatrist collaborative patient education resulted in a statistically significant improvement in patient’s medication adherence and health-related quality of life compared to the usual care group. Overall, the study results indicated that pharmacist-psychiatrist collaborative care can significantly improve the patient’s medication adherence and health-related quality of life.

## Data Availability

The original contributions presented in the study are included in the article/supplementary material. Further inquiries can be directed to the corresponding author.
